# Association between experiences of discrimination and mental health among persons with disabilities in Canada during the COVID 19 pandemic

**DOI:** 10.1007/s44192-025-00351-x

**Published:** 2025-12-13

**Authors:** Sulemana Ansumah Saaka, Christa Sato

**Affiliations:** 1 Department of Geography and Environment, Faculty of Social Science, University of Western Ontario, London, Canada; 2 Faculty of Social Work, University of Toronto, Toronto, Canada; 3 School of Social Work, University of Northern British Columbia - NW Campus, Canada

**Keywords:** Canada, Discrimination, Mental health, PWDs, Social connections

## Abstract

Discrimination against Persons with disabilities (PWDs), a pervasive issue that predates COVID-19, was reportedly magnified and manifested in both overt and subtle ways during the pandemic with implications for the mental health (MH) of PWDs. Nonetheless, far less work has focused on how experiences of discrimination affected the MH of PWDs during the pandemic in Canada. By utilizing data from the 2022 Canadian General Social Survey (*N* = 13,347), a subset of PWDs, for cross-sectional analyses of the impact of discrimination on mental health (MH) of PWDs, the results indicate that individuals who experienced discrimination based on their physical/mental disability status, physical appearance, and sex, all significantly reported lower odds of High Self-rated Mental Health (HSRMH) relative to those who did not experience these forms of discrimination. Those with multiple disability counts further reported lower odds of HSRMH relative those with only one disability count. On the contrary, having strong social connections, correlated more with HSRMH. Moreover, age, marital status, educational attainment, immigration status, and province of residence significantly predicted the MH of PWDSs in the study context. Thus, disability-related discrimination adversely affects the MH of PWDs in Canada, particularly, those with multiple disabilities.

## Introduction

COVID-19 has profoundly impacted societies worldwide, exacerbating existing inequities and vulnerabilities, as well as introducing new challenges among marginalized populations. Persons with disabilities (PWDs) have faced disproportionate challenges during this public health crisis, as pre-existing structural barriers and social stigmas were magnified [1]. In Canada, where about 6.2 million people—or 22% of the population have some form of disability [2], the pandemic underscored the interplay between discrimination, social connections, and MH outcomes within this community. As governments implemented stringent COVID-19 containment measures including lockdowns and physical distancing mandates, the implications for PWDs extended beyond physical health risks to encompass profound social and psychological consequences [3, 4].

Discrimination against PWDs is a pervasive issue that predates COVID-19, rooted in societal attitudes that often marginalize and exclude PWDs. These discriminatory practices manifested in both overt and subtle ways since the COVID-19 crisis. For example, early triage protocols in some healthcare systems throughout the pandemic have prioritized non-disabled individuals, raising ethical concerns about equitable access to care [5]. Such systemic biases contributed to heightened feelings of social devaluation and alienation among PWDs, thereby intensifying MH challenges among this group. Moreover, although strong social connections play a critical role in promoting mental well-being by serving as a buffer against stress and isolation [6], the pandemic-related restrictions significantly disrupted the social networks of PWDs [7], most of whom often rely on in-person support systems such as caregivers, family support, community programs, and peer groups. The abrupt transition to virtual platforms posed accessibility challenges, as many digital tools and communication platforms were not designed with inclusivity in mind [8]. This digital divide left many PWDs disconnected from essential resources and social interactions, exacerbating feelings of loneliness and psychological distress [8]. For individuals with sensory or cognitive disabilities for instance, navigating these new modes of communication may prove particularly difficult, further isolating them from their communities. In a scoping review, Saldanha et al., [8] further observed that in the context of the public health emergency, PWDs have been historically marginalized with dire implications for their vulnerability, deprivations, and prejudices and stigmas that influence decision-making in healthcare and exacerbate preexisting inequalities, making this group more susceptible to illness and lack of social protection.

The intersection of discrimination and disrupted social connections has had profound implications for the MH of PWDs throughout the pandemic. Studies conducted in Canada [9–12] and globally have highlighted elevated rates of anxiety, depression, and stress among the general population during the pandemic with less focus on PWDs [13]. Thus, understanding the experiences of PWDs throughout the COVID-19 pandemic can illuminate the complex interplay between societal discrimination, the attrition of social connections, and MH outcomes. This inquiry will not only unearth the unique vulnerabilities faced by PWDs but also highlights opportunities to reimagine social inclusion and MH services in a post-pandemic world. In a world where global pandemics are becoming increasingly common, addressing the unique challenges of PWDs is essential to fostering resilience and ensuring that no one is left behind in the post-pandemic recovery process.

### Theoretical underpinning of study

In evaluating the impact of discrimination on MH of PWDs in Canada, this study is underpinned by the social model of disability (SMD) [14, 15] and the socio-ecological framework [16]. The SMD posits that disability is not exclusively a result of the individual's physical/mental impairments, but also, a condition shaped by the social, cultural, political, and economic factors that influence the experiences of PWDs [17]. Important distinctions between *impairment* (i.e., a functional limitation caused by physical, mental, or sensory defect), and *disability* (i.e., a loss or limitation of opportunities to equally partake in the normal life of a community due to both physical and social barriers) are thus made by the SMD [15, 18, 19]. SMD places disability squarely within society [18, 20], denoting that the impairment of PWDs is not the sole cause of their inability to fully function, but rather, society’s failure to accommodate the needs of PWDs in social settings. SMD thus draws attention to the dynamic socio-environmental conditions in which *disability* is constructed [21–23].

The theoretical constructs of SMD is complemented by socio-ecological perspective on how disabilities are shaped by the broader social and physical surroundings [23]. Hence this study also draws on insights from the socio-ecological framework which emphasizes the complex interaction between individuals and their social environments, as well as the multi-layered influence of socio-ecological systems in shaping human development and wellbeing [16]. The socio-ecological framework holds that microsystems such as family, schools, peer groups, and neighborhoods, form the immediate environment that people interact with. The individual’s interactions with, and the interconnections between these microsystems, external environments (such as the COVID-19 policies that indirectly influence the individual but do not involve the individual directly), and macrosystems (i.e., cultural values, societal norms) have an overarching influence on their wellbeing [16]. Within the context of the COVID-19 for instance, macro environmental factors such as the closure of essential services, travel bans, lockdowns, and other COVID-19 restrictions have been shown to have MH ramifications [10, 24–27]. For instance, a prior study that examined the impact of the COVID-19 pandemic on MH of PWDs, indicated that external environmental factors (including COVID-19 policies) during the pandemic may have heightened pre-existing risks and vulnerabilities of PWDs by limiting their access to support services such as in-person healthcare, personal assistance, and other community support programs [28]. Thus, similar to the SMD’s postulation (i.e., disability is not the sole cause of one’s inability to fully function, but rather, society’s failure to accommodate the needs of PWDs in social settings), the socio-ecological environment undoubtedly plays a significant role in the shaping wellbeing and full potentials of PWDs. Thus, to understand the unique challenges of PWDs during the COVID-19 and the impact on their MH, this study draws its theoretical inspirations from both the SMD and socio-ecological theory.

## Methods and materials

### Data collection and sampling frame

Data collection for the 2020 General Social Survey (GSS) on Social Identity (SI) took place from August 17, 2020, to February 7, 2021. Data were collected electronically as a self-completed questionnaire (rEQ) as well as via computer assisted telephone interviews (iEQ) where respondents were interviewed in the official language of their choice. Proxy interviews were not permitted. Interviewers were trained by Statistics Canada staff in telephone interviewing techniques using the iEQ, as well as in survey concepts and procedures. Interviewers were instructed to make all reasonable attempts to contact the selected member of the household, 15 years of age or older. The survey used the Dwelling Universe File (DUF), a file produced at Statistics Canada, as the sampling frame. This was done to produce quality estimates at the provincial level, and to facilitate an initial contact by mail for the invitation to complete the questionnaire electronically. This sampling frame allows for up to three telephone numbers to allow for telephone follow-up with a household, including landline and cellular telephone numbers. Dwellings that were identified as vacant at the time the sampling frame was created were excluded from the sample frame. Dwellings that had neither a mailing address nor an associated telephone number were also excluded from the sample frame, as they could not be contacted by any of the survey collection modes. However, the survey estimates were weighted to include persons living in these dwellings.

The sample design for the 2020 GSS was a stratified two-stage random sample. The provinces formed the strata. In the first stage, households were selected randomly, and in the second stage, one person was selected from within the household using the age-order selection method. The selection algorithm was based on the number of eligible members in the household and the ordered age of each member. A letter was sent to the selected household, and a household member was selected, via the instructions provided in the letter, to complete the electronic questionnaire. The selected person was invited to complete the questionnaire by accessing it online and entering a secure access code (SAC) provided in the letter. Age-order selection was also used for computer-assisted telephone interviews (iEQ respondents). Selection was done with the interviewer. The instructions in the letter, as well as the selection made with the interviewer, were consistent for the same sampled household to ensure that the same person was selected to participate in the survey for a given household, regardless of the collection mode used to complete the questionnaire. 97.3% of the selected dwelling emerged as eligible households. To be eligible, a household had to include at least one person 15 years of age or older. Thus, on consented and qualified individuals were included in the survey. During data collection, households that did not meet the eligibility criteria were terminated after an initial set of questions that determined eligibility. The expected sample size (i.e., the expected number of respondents) for Cycle 35 SI was 30,000, while the actual number of respondents was 34,044. The overall response rate was 40.3%.

### Measures

The dependent variable for this study is Self-Rated Mental Health (SRMH). It is derived from a question where participants were asked “In general, how is your mental health?” with “Excellent”, “Very Good”, “Good”, “Fair”, and “Poor” as the response categories. Based on earlier studies [11, 28, 29] “Excellent” “Very Good” and “Good” were coded as High self-rated mental health (HSRMH), the dependent variable for this study. Independent variables: Theoretically relevant variables were included in the models as predictors of HSRMH among PWDs. The general question preceding each discrimination-related measure was “*Since the beginning of the COVID-19 pandemic, have you experienced discrimination or been treated unfairly by others in Canada because of any of the following?*”. Specifically, participants were questioned whether they experienced discrimination based on their language, ethnicity, race, religion, sex, physical appearance, and physical/mental disability status. All these variables were categorical measures (response categories included “Yes”, “No”, and “Not stated”). Given that the focus of this paper is on experiences of discrimination and how that is associated with mental health of participants, we dichotomized the responses (“No/Not stated” and “Yes”) to predict the relationship between experiences of discrimination and the self reported mental health status of those who responded “Yes”, relative to those who responded “No/Not Stated”. These discrimination-related variables capture structural and interpersonal discrimination, reflecting the SMD’s emphasis on societal barriers rather than individual impairments. For instance, experience of discrimination based on physical/mental disability status, physical appearance, race/color, ethnicity/culture, religion, and sex, are directly tied to SMD. These measures assess societal exclusion of PWDs, a reflection of stigma associated with one’s disability status, as well as the intersectionality of discrimination based on multiple identities.

Also, the specific types of disabilities that were measured in the 2020 GSS included mental health disability, seeing disability, hearing disability, mobility disability, flexibility disability, dexterity disability, Pain-related disability, learning disability, developmental disability, and memory disability. Although these variables were not directly included in our analysis, the variable “Disability Status” is a derived variable from the afore mentioned disability types, which indicates whether a person has a disability. Thus, a person is defined as having a disability if he or she has one or more of the following types of disability: seeing, hearing, mobility, flexibility, dexterity, pain-related, learning, developmental, memory, mental health-related, or unknown. It is important to note that this variable (Disability Status) has no residual "Not Stated" category. Persons who do not have at least one disability, as defined by the specific disability status variables, are considered not to have a disability. Thus, from the 2020 Canadian General Social Survey (*N* = 32,029), our analyses are based solely on a subset of PWDs (*N* = 13,347). Focusing on a subset of only PWDs first and foremost, reduces heterogeneity within the study population and allow for a more precise examination of the association between discrimination experiences and mental health of PWDs. Also, given that the 2020 Canadian General Social Survey represent a highly diverse group whose experiences vary widely, analyzing a more homogeneous subset helps ensure that observed associations are not obscured by this variability. Additionally, it improves the internal validity of comparisons and allows for clearer interpretation of patterns of discrimination and the mental-health impacts. Disability count (i.e., the number of disabilities an individual have) was included as a disability-related variable in our models for analysis.

Moreover, variables on social connections included the numbers of close relatives and friends that PWDs had. Social connection variables (Microsystem Level) under the socio-ecological framework highlights the immediate social environments as a critical factor to mental health (MH). For example, the number of close relatives/friends is a measure of social capital, where fewer connections may exacerbate MH risks amidst widespread discrimination and societal barriers. Sociodemographic variables included age, gender, marital status, educational attainment, and immigration status. These sociodemographic variables (Individual & Mesosystem Levels) control for personal and intersecting societal influences. In terms of age for instance, older PWDs may face compounded discrimination (e.g., ageism & ableism). Likewise, gender norms may shape disability experiences (e.g., gendered care expectations), while one’s marital status may shape their social/emotional support or isolation. Moreover, exosystem/macrosystem factors such as immigration status (Canadian-born, immigrant, refugee) can shape different levels of marginalization or systemic exclusion. Geographically, the province of residence was also controlled for in the models. At the macrosystem level, province of residence captures regional policy/cultural differences (e.g., healthcare access, disability supports), aligning with the socio-ecological framework macrosystems.

### Analytical approach

We employed descriptive statistics to provide an overview of the sample characteristics (Table 1). Both bivariate and multivariable logistic regression models were further utilized to examine the association between all independent variables and the outcome variable. However, we observed that the bivariate results did not differ from the multivariate results. Thus, our analysis was limited to the multivariate results (Table 2). Two multivariable regression models were conducted where we controlled for all variables in model 1 to better understand the effect of the disability-specific variables in model 2. Results for the multivariable regression models are reported in Adjusted Odds Ratios (AOR) where significant odds ratios above one (AOR > 1) indicate higher likelihood of HSRMH, while odds ratios below one (AOR < 1) indicate lower likelihood of HSRMH. All statistical data analyses were conducted using Stata version 18. The use of the 2020 GSS for this analysis is predicated on the fact that aside the dataset being nationally representative, the period of data collection (during COVID-19 pandemic) providing contextual relevance to the focus of the current study, while prior studies further suggest that the mental health challenges of PWDs were exacerbated during the pandemic [28, 30]. Also, while similar studies have utilized the Canadian Housing Survey (CHS), the GSS.

**Table 1:** Demographic characteristics of study participants

Variable	Frequency (%)
*Disability Count*	
One	6264(46.93)
Two	4772(35.75)
Three or more	2311(17.31)
*Gender*	
Male	5786(43.35)
Female	7561(56.65)
*Age group*	
15 to 24 years	894(6.70)
25 to 34 years	1477(11.07)
35 to 44 years	1993(14.93)
45 to 54 years	2099(15.73)
55 to 64 years	2695(20.19)
65 to 74 years	2526(18.93)
75 years and over	1663(12.46)
*Marital Status*	
Married	5491(41.14)
Living in common-law	1134(8.50)
Widowed	1271(9.52)
Separated	543(4.07)
Divorced	1577(11.82)
Single/never married	3303(24.75)
Not stated	28(0.21)
*Educational attainment*	
Less than diploma	1842(13.80)
Diploma	7313(54.79)
Bachelor	2437(18.26)
Post Bachelor	1460(10.94)
Not stated	295(2.21)
*Landed Immigrant*	
No	8528(63.89)
Yes	4819(36.11)
*Province of residence*	
Newfoundland and Labrador	430(3.22)
Prince Edward Island	481(3.60)
Nova Scotia	742(5.56)
New Brunswick	572(4.29)
Quebec	2496(18.70)
Ontario	4465(33.45)
Manitoba	726(5.44)
Saskatchewan	618 (4.63)
Alberta	1253(9.39)
British Columbia	1564(11.72)

**Table 2:** Multivariable regression results for experience of discrimination and Self-reported mental health of PWDs in Canada during COVID-19

Variables	Model 1		Model 2	
	AOR (SE)	95% CI	AOR (SE)	95% CI
*Discriminated based on Physical appearance (Ref: No/Not Stated)*				
Yes	0.677(0.043) ***	0.597 0.767	0.815(0.054) **	0.715 0.928
*Discriminated based on Language (Ref: No/Not Stated)*				
Yes	0.877(0.069)	0.751 1.024	0.923(0.074)	0.788 1.082
*Discriminated based on Ethnicity/culture (Ref: No/Not Stated)*				
Yes	1.013(0.081)	0.866 1.185	1.015(0.083)	0.864 1.192
*Discriminated based on Race/color (Ref: No/Not Stated)*				
Yes	1.221(0.095) *	1.048 1.422	1.207(0.096) *	1.032 1.411
*Discriminated based on Religion (Ref: No/Not Stated)*				
Yes	1.121(0.104)	0.934 1.346	1.275(0.122) *	1.057 1.539
*Discriminated based on Sex (Ref: No/Not Stated)*				
Yes	0.727(0.050) ***	0.634 0.833	0.772(0.055) ***	0.670 0.889
*Number of relatives respondent feels close to (Ref: None)*				
1	1.459(0.146) ***	1.199 1.776	1.430(0.147) ***	1.169 1.751
2	1.664(0.154) ***	1.387 1.998	1.596(0.152) ***	1.324 1.926
3	1.837(0.178) ***	1.519 2.223	1.729(0.172) ***	1.422 2.102
4	1.996(0.206) ***	1.631 2.444	1.876(0.198) ***	1.524 2.308
5	2.169(0.234) ***	1.755 2.680	2.078(0.230) ***	1.672 2.582
6	2.199(0.279) ***	1.714 2.822	2.115(0.276) ***	1.638 2.733
7	2.673(0.448) ***	1.923 3.715	2.465(0.421) ***	1.763 3.448
8	2.711(0.442) ***	1.968 3.733	2.756(0.463) ***	1.983 3.831
9	2.691(0.716) ***	1.596 4.536	2.674(0.731) ***	1.564 4.572
10 to 19	2.845(0.346) ***	2.241 3.611	2.672(0.332) ***	2.094 3.409
20 to 29	2.892(0.719) ***	1.776 4.709	2.830(0.718) ***	1.720 4.656
30 or more	1.833(0.550) *	1.018 3.301	1.966(0.604) *	1.076 3.592
*Number of friends respondent feels close to (Ref: None)*				
1	1.233(0.109) *	1.036 1.467	1.199(0.109) *	1.003 1.434
2	1.631(0.136) ***	1.385 1.922	1.521(0.130) ***	1.285 1.800
3	1.765(0.156) ***	1.484 2.099	1.641(0.1490) ***	1.373 1.961
4	1.949(0.190) ***	1.610 2.361	1.750(0.174) ***	1.439 2.128
5	2.188(0.215) ***	1.803 2.655	1.972(0.199) ***	1.617 2.405
6	2.443(0.298) ***	1.922 3.105	2.266(0.283) ***	1.773 2.896
7	2.432(0.438) ***	1.708 3.463	2.125(0.392) ***	1.479 3.053
8	2.461(0.399) ***	1.790 3.384	2.206(0.366) ***	1.593 3.054
9	1.891(0.556) *	1.062 3.368	1.877(0.566) *	1.039 3.392
10 to 19	3.078(0.362) ***	2.444 3.877	2.808(0.339) ***	2.216 3.558
20 to 29	2.739(0.680) ***	1.683 4.457	2.548(0.647) ***	1.549 4.192
30 or more	3.975(1.586) ***	1.818 8.690	3.579(1.444) **	1.623 7.894
Not Stated	2.084(0.859)	0.928 4.678	1.753(0.728)	0.776 3.957
*Gender (Ref: Male)*				
Female	0.915(0.045)	0.831 1.008	0.947(0.047)	0.858 1.045
*Age group (Ref: 15 to 24 years)*				
25 to 34 years	1.314(0.125) **	1.089 1.584	1.474(0.144) ***	1.217 1.785
35 to 44 years	1.673(0.162) ***	1.383 2.023	2.060(0.205) ***	1.694 2.504
45 to 54 years	2.874(0.289) ***	2.358 3.501	3.865(0.403) ***	3.149 4.744
55 to 64 years	3.963(0.400) ***	3.251 4.832	5.786(0.612) ***	4.702 7.120
65 to 74 years	6.699(0.753) ***	5.374 8.352	9.897(1.160) ***	7.865 12.454
75 years and over	7.233(0.959) ***	5.578 9.380	11.561(1.599) ***	8.814 15.162
*Marital Status (Ref: Married)*				
Living in common-law	0.826(0.073) *	0.695 0.983	0.835(0.075) *	0.699 0.997
Widowed	0.641(0.068) ***	0.519 0.791	0.676(0.073) ***	0.546 0.838
Separated	0.588(0.066) ***	0.471 0.734	0.622(0.072) ***	0.495 0.780
Divorced	0.585(0.046) ***	0.500 0.684	0.635(0.052) ***	0.540 0.746
Single/never married	0.563(0.036) ***	0.496 0.638	0.609(0.040) ***	0.535 0.693
Not stated	0.446(0.210)	0.177 1.125	0.456(0.217)	0.179 1.160
*Educational attainment (Ref: Less than high school)*				
Diploma	1.235(0.087) **	1.075 1.419	1.169(0.084)	1.015 1.348
Bachelor	1.440(0.123) ***	1.217 1.705	1.230(0.108)	1.035 1.462
Post Bachelor	1.548(0.154) ***	1.273 1.884	1.276(0.130) *	1.043 1.560
Not stated	1.115(0.179)	0.812 1.529	1.193(0.198)	0.861 1.653
*Landed Immigrant (Ref: No)*				
Yes	1.286(0.072) ***	1.151 1.437	1.241(0.071) ***	1.107 1.390
*Province of residence (Ref: Newfoundland and Labrador)*				
Prince Edward Island	1.014(0.172)	0.726 1.417	0.954(0.166)	0.678 1.343
Nova Scotia	0.978(0.151)	0.722 1.326	1.012(0.161)	0.741 1.382
New Brunswick	1.188(0.199)	0.855 1.651	1.187(0.204)	0.848 1.663
Quebec	2.131(0.298) ***	1.619 2.805	1.996(0.286) ***	1.507 2.643
Ontario	1.063(0.141)	0.819 1.380	1.047(0.1425	0.802 1.367
Manitoba	1.104(0.173)	0.811 1.503	1.076(0.173)	0.784 1.475
Saskatchewan	1.153(0.187)	0.838 1.586	1.078(0.179)	0.778 1.494
Alberta	1.113(0.161)	0.837 1.480	1.060(0.157)	0.791 1.419
British Columbia	1.107(0.158)	0.837 1.466	1.076(0.157)	0.808 1.434
*Disability Count (Ref: one disability)*				
Two			0.544(0.029) ***	0.490 0.606
Three or more			0.289(0.019) ***	0.253 0.331
*Discriminated based on Physical/mental disability (Ref: No/Not Stated)*				
Yes			0.520(0.047) ***	0.435 0.622
Goodness of model fit measures	Pseudo R^2^ = 0.136, Number of observations = 13,347		Pseudo R^2^ = 0.169, Number of observations = 13,347	

## Results

### Descriptive statistics of study participants

Table 1 presents the results for descriptive statistics of study participants. From the results, the majority (56.65%) were females, had a diploma (54.79%) and were Canadian born (63.89%). Also, a significant proportion of them had at least one disability count (46.93%), were within the age brackets of 55 to 64 years (20.19%), married (41.14%) and resided in Ontario (33.45%).

#### Multivariable analyses of the impact of discrimination on mental health of PWDs in Canada

Results for multivariate analyses are presented in Table 2. We controlled for disability-related variables in our analysis. Thus, while disability-related variables were excluded from model 1, they were introduced in model 2 to help ascertain their impact on mental health. After introducing the disability-related variable in model 2, the results mirrored that of model 1. However, there was an observed improvement in the model fitness as the R-squared value increased from 0.136 in model 1 to 0.169 in model 2, suggesting more robustness in model 2, and that disability itself and disability count has significant effect on the mental health of PWDs.

Thus, given that the results of model 2 mirrored that of model 1, and emerged more robust in terms of model fitness, our analysis and discussions are predicated on model 2. From the results in model 2, PWDs who have been discriminated based on their physical/mental disability status (AOR = 0.520; *p* < 0.001), physical appearance (AOR = 0.815; *p* < 0.01), and sex (AOR = 0.772; *p* < 0.01), all significantly reported lower odds of HSRMH relative to those who did not experience these forms of discrimination. Moreover, relative to those with only one disability count, those with two disability counts (AOR = 0.544; *p* < 0.01) as well as those with three or more disability counts (AOR = 0.289; *p* < 0.01), significantly reported lower odds of HSRMH. However, those who experienced discrimination based on Race/color (AOR = 1.207; *p* < 0.05) and religion (AOR = 1.275; *p* < 0.05), reported higher odds of HSRMH. Also, age and social connection in terms of the number of close friends and relatives, positively and significantly correlated with HSRMH (See Table 2). Furthermore, compared to those who were married, those who were living in common-law (AOR = 0.835; *p* < 0.05), widowed (AOR = 0.676), separated (AOR = 0.622; *p* < 0.001), divorced (AOR = 0.635; *p* < 0.001), single/never married (AOR = 0.609; *p* < 0.001), all significantly reported lower odds of HSRMH. Additionally, post-bachelor’s degree holders (AOR = 1.276; *p* < 0.05) and landed immigrants (AOR = 1.241; *p* < 0.001), significantly reported higher odds of HSRMH than those with less than a diploma and those who are Canadian born, respectively. Provincial disparities were further observed as the residents of Quebec (AOR = 1.996; *p* < 0.001) significantly reported higher odds of HSRMH relative the residents of Newfoundland and Labrador (See Table 2).

## Discussion

Guided by the social model of disability (SMD) [1, 2] and the socio-ecological framework [3], we evaluated the impact of discrimination on the mental health of PWDs in Canada. The results showed that PWDs, particularly those with multiple disability count, and those who experienced discrimination, all reported lower odds of HSRMH relative to those with just one disability count and those who did not experience discrimination, respectively. On the contrary, those who had strong social connections in terms of number of close relatives and friends, reported higher odds of HSRMH. Moreover, age, marital status, educational attainment, immigration status, and the province of residences significantly predicted HSRMH in the study context. These findings are discussed in the ensuing paragraphs.

Persons with multiple disability counts, those who experienced discrimination based on their physical or mental disability status, physical appearance, and sex all reported lower odds of HSRMH compared to those who did not face such discrimination. Our finding corroborates an Australian study where disability-based discrimination was found to be strongly associated with psychological distress [31]. A related study in the United States established that exposure to the pandemic-related stressor was associated with greater discrimination, which increased the psychological distress of PWDs relative to people without disabilities [32]. Undoubtedly, social exclusion and marginalization have a detrimental effect on the mental well-being of PWDs. Discrimination in various forms may contribute to feelings of isolation, stress, and reduced self-worth, all of which can negatively impact MH. The lower odds of HSRMH among those with multiple disability counts further underscores the compounding effect of both discrimination and the increased burden of disability itself on their MH. The combined challenges of facing discrimination and managing the burden of multiple disabilities likely created more complex MH experiences, leading to poorer MH outcomes. This finding not only collaborate the SMD’s emphasis on societal barriers rather than individual impairments but also highlight the critical need for addressing both systemic discrimination and the unique MH needs of individuals with multiple disabilities to improve their overall well-being.

Although individual and mesosystem levels factors like age may have personal and intersecting societal influences on experiences of discrimination (e.g., ageism & ableism), we found that age was positively associated with HSRMH in the context of this study. As individuals age, they may gain more life experience and coping mechanisms, which could contribute to their resilience and improved mental well-being during public health crisis. Besides, the prevalence of most behavioral and anxiety disorders (e.g., mood swings and substance use) are reportedly more apparent during adolescence and early adulthood, as do psychotic disorders [33]. Moreover, although the prevalence of disability increases with age in Canada, MH related disabilities are particularly more pronounced among youth aged 15 to 24 years [2]. There is also a stark prevalence of ableism and related discrimination among young adults with disabilities than older age cohorts [34]. Studies on help-seeking behaviors further suggest that older adults tend to exhibit more favorable intentions to seek help for their mental health than younger adults [35]. Collectively, these factors tend to shape different levels of vulnerabilities among different age groups.

As posited by the socio-ecological framework, the individuals’ interaction with microsystems such as family, schools, peer groups, and neighborhoods influences their wellbeing. Hence, it is therefore not surprising that there was a positive relationship between social connection and HSRMH in this study context. A strong social support networks (i.e., the number of close friends and relatives) plays a crucial role in enhancing MH as it provides emotional support, reduce feelings of isolation, and thus offer resources to cope with stress, ultimately fostering a better sense of self-rated MH. For instance, a Canadian-based study uncovered that beyond COVID-19 stress itself, more family time was related to less depression relative to virtually connection during the pandemic [36]. While our findings are suggestive that both age and strong social ties are important factors that contribute to better MH outcomes, it is worth emphasizing the two factors are interconnected as aging without a strong social connection may lead to loneliness with detrimental impacts on one’s MH. Closely related to social connection, and consistent with prior studies [33], those who were married reported higher odds of HSRMH compared to those in common-law partnership, the widowed, the separated, divorced individuals, and singles/never married. The individuals’ interaction with microsystems including their spouses in their immediate environment, undoubtedly shapes their daily life experiences and growth. Thus, the positive relationship between being in a marital relationship and having HSRMH in this context, is suggests that a good marital relationship may provide emotional stability, support, and a sense of belonging, all of which can contribute positively to MH. The presence of a supportive partner may reduce feelings of loneliness and stress, which are known to negatively affect mental well-being. Although studies elsewhere demonstrated that experience of intimate-partner violence as well as the socioeconomic status of married individuals play a major role in the state of mental wellbeing [37], PWDs who are in non-marital relations, and lack strong social ties, must be prioritized in programs for social inclusion and the promotion of mental health.

The study further unveiled that, individuals with a post-bachelor’s degree had higher odds of HSRMH compared to those with less than a diploma, a finding that also aligns with the socio-ecological framework’s postulation that interactions with microsystems like schools can influence life experiences (including discrimination) and the wellbeing of individuals. Studies have highlighted the crucial role of educational institutions in offering peer health educator programs that instil students with stress-coping skills with positive impact on their MH [38]. Other studies suggest that psychosocial resources such as self-efficacy, optimism, sense of control, social support, coping strategies or resilience, emotional intelligence are less apparent among people with a lower level of educational attainment, hence their inability to optimally cope with adversities [39]. Such internal qualities, traits, or capabilities are however crucial for the resilience and mental health, especially during public health crises such as the COVID-19 pandemic [11]. Individuals with advanced education may also have more stable employment, better access to healthcare, and stronger coping mechanisms, which can positively impact their mental health. This finding thus highlights the crucial need for disability inclusion policies to prioritize individuals with lower levels of educational attainment.

Furthermore, landed immigrants reported higher odds of HSRMH compared to non-landed immigrants. Although landed immigrants may face uncertainty regarding their residency status, which can cause stress, anxiety, and limitations on access to essential resources with negatively implications for their MH outcomes, evidence suggests that migration related challenges does not exclusively lead to mental distress [40]. In other words, the risk of developing a mental illness is not contingent on migration itself, but determined largely by traumatic experiences involved in the migration process, the level of vulnerability of the immigrants themselves, as well as the level of stressors or hostility in the host country. A prior Canada based study suggest that the positive association between being an immigrant and having higher likelihood of better mental health could be due to the g the “healthy immigrant effect” where immigrants enjoy better health than native-born people albeit they experience a decline in health over time in their destination areas [28].

Geographically, provincial disparities were observed with residents of Quebec significantly reporting higher odds of HSRMH compared to those living in Newfoundland and Labrador. Related studies in Canada have attributed such disparities in MH variations in exposure to MH stressors, access to disability-friendly healthcare facilities, social support systems, provincial policies and funding for MH programs targeting PWDs, and variations in COVID-19 containment measures [11, 41]. The individual’s interactions with, and the interconnections between these microsystems and external environments (such as the Covid-19 policies), undoubtedly have an overarching influence on their wellbeing [16]. Within the context of the COVID-19 for instance, macro factors including the closure of essential services, imposition of travel bans, lockdowns, and other Covid restrictions have been shown to have MH ramifications [10, 24–27]. Overall, the observed provincial differences in MH outcomes underscores the need for province-specific interventions that addresses the MH needs of PWDs.

### Study limitations

We report some noteworthy limitations of the study. First, the quantitative and cross-sectional nature of the study limits our ability to causal conclusion. Thus, future studies can benefit from a mixed-method approach. Second, given the self-reported nature of the study variables, the responses of participants may be subject to response and social desirability biases, possibly influencing the findings. Also, the current study may not be exhaustive of all contextually relevant variables that contributed to the observed outcomes. Notwithstanding these limitations, our results provide valuable contributions to the broader literature on the social inclusion and mental wellbeing of PWDs.

## Conclusions and recommendations

From our findings, younger age cohorts with multiple disabilities, weak social connection, and lower educational attainment, are more vulnerable to poor MH outcomes in Canada, especially during public health crisis. Hence, policy intervention must target these groups at the individual, community, provincial, and national level. The findings thus highlight the need for inclusive public health policies that address the compounded disadvantages that PWDs may face in future public health crises. Given that discrimination based on disability, appearance, race, religion, and sex significantly lowers mental health outcomes for PWDs, public health strategies should prioritize anti-discrimination frameworks and mental health support for marginalized groups, ensuring equitable access to care.

Additionally, the increased vulnerability of individuals with multiple disabilities demands tailored interventions that account for the complexity of their needs. This could include development of a specialized community health program that combines physical, mental, and social care. For instance, a healthcare center could offer multidisciplinary teams consisting of physical therapists, mental health professionals, social workers, and disability support specialists. These teams can work together to create personalized care plans that address the individual's unique needs, such as mobility support, mental health counseling, and social connection strategies. Additionally, tailored technology-driven solutions like telehealth services could be implemented during future public health crisis to provide more accessible and consistent care for individuals with multiple disabilities, particularly in remote areas where these services may not be readily available. This could include virtual check-ins with specialists, and virtual peer support groups to foster social inclusion. The positive correlation between social connection and mental health further emphasizes the importance of community-based support programs. Also, provincial disparities suggest that localized health policies should be adapted to address specific regional needs, particularly for PWDs in less supportive provinces. Moreover, the findings underscore the need for more public education and awareness creation about discrimination and the MH implications on PWDs. Specifically, schools, social work agencies, health facilities and programs that focus on the mental health of PWDs should integrate lessons from the pandemic into their curricular and training of health and social care professionals. Lastly, it is crucial to increase capacity building among service providing agencies that work at the intersections of disability, health and social care, for ensuring equitable access to MH resources among PWDs post-pandemic.

## Data Availability

The datasets used and/or analysed during the current study are available from the corresponding author on reasonable request.
